# Stiffness of the microenvironment upregulates ERBB2 expression in 3D cultures of MCF10A within the range of mammographic density

**DOI:** 10.1038/srep28987

**Published:** 2016-07-07

**Authors:** Qingsu Cheng, Cemal Cagatay Bilgin, Gerald Fonteney, Hang Chang, Matthew Henderson, Ju Han, Bahram Parvin

**Affiliations:** 1 1664 N Virginia Street, Department of Electrical and Biomedical Engineering, University of Nevada, Reno, Reno NV, 89503, USA; 2 Lawrence Berkeley National Laboratory, 1 Cyclotron Road, MS-977, Life Science Division, Berkeley CA, 94720, USA

## Abstract

The effects of the stiffness of the microenvironment on the molecular response of 3D colony organization, at the maximum level of mammographic density (MD), are investigated. Phenotypic profiling reveals that 3D colony formation is heterogeneous and increased stiffness of the microenvironment, within the range of the MD, correlates with the increased frequency of aberrant 3D colony formation. Further integrative analysis of the genome-wide transcriptome and phenotypic profiling hypothesizes overexpression of ERBB2 in the premalignant MCF10A cell lines at a stiffness value that corresponds to the collagen component at high mammographic density. Subsequently, ERBB2 overexpression has been validated in the same cell line. Similar experiments with a more genetically stable cell line of 184A1 also revealed an increased frequency of aberrant colony formation with the increased stiffness; however, 184A1 did not demonstrate overexpression of ERBB2 at the same stiffness value of the high MD. These results suggest that stiffness exacerbates premalignant cell line of MCF10A.

The extracellular matrix (ECM) plays important roles including regulating structure and function of the cell and cellular organization, and in achieving homeostasis^1^. The ECM provides structural support for the cell growth, regulates the local pH and concentration of growth factors and receptors, and is a dynamic entity that can go through remodeling in response to stress and environmental factors^2^. The dynamic reciprocity model suggests that the interactions of chemical and physical components in the ECM with the cell surface receptors have a profound impact on cellular morphology, colony organization, and gene expression^3^,^4^. These changes, in turn, remodel the ECM until homeostasis is met. A predominant component of the ECM is collagen, which has been shown in the breast tumor microenvironment to be heavily cross-linked. Studies in mice^5^ have shown that tumor regions are consistently stiffer than the normal tissue. Increased cross-linking is manifested through increased stiffness of the microenvironment, which alters the tissue mechanics and is the basis for breast self-examination. human mammary epithelial cells (HMEC) can sense and respond to the local mechanical forces by reorganizing the cytoskeleton and altering a number of pathways, known as mechanotransduction signaling, in a force-dependent manner^6^,^7^, with the integrin family playing a key role in sensing and transmitting force from the ECM^8^. The integrin family is involved in cell adhesion, and regulates a number of cellular functions that are involved in cancer initiation, progression, and metastasis. For example, aberrant expression of β4-integrin is associated with breast cancer progression, and the loss of β4-integrin down-regulates the activity of the PI3K/Akt pathway^9^, which is a known pathway in ERBB2 expression^10^,^11^,^12^. More recently, it has been shown that TWIST1 is also an essential mediator for epithelial-meshenchymal transition (EMT) in response to increased stiffness^13^.

This paper has two overarching goals. First, it advances the current understanding of the role of increased stiffness on colony organization and its molecular endpoints, within the range of mammographic density (MD), through detailed confocal microscopy and molecular profiling. MD represents the density of connective and epithelial tissue in breast^14^, and is a known and important risk factor for breast cancer. Low to high MD are currently documented during mammographic screening, and classified using a 1–4 rating based on the Breast Imaging, Reporting & Data System (BIRADS)^15^. Using this system, a rating of 1 denotes 25% dense tissue (almost entirely fat), a 2 denotes 26–50% dense tissue (scattered fibroglandular densities), 3 denotes 51–75% dense tissue (heterogeneously dense), and a 4 signifies more than 75% dense breast tissue (extremely dense). Typically, women with a score of 3–4 are designated as having dense breasts. Breast density imparts a greater risk than age of menopause^16^, parity, or family history. Only age and BRCA mutation status are associated with a larger relative risk of breast cancer compared to breast density^17^,^18^. Current studies suggest that women with highest density have a 4- to 6-fold increase risk of breast cancer compared to women with the lowest density^19^,^20^,^21^. High breast density is prominent in stromal fibrosis, and has a higher level of collagen, which not only alters the stromal protein expression, but also increases the stromal stiffness^22^,^23^. Second, measuring the stiffness of the MD provides the basis for engineering matrices for 3D cultures and profiling colony formation with embedded protocol^24^. The embedded protocol assures that every cell in the colony receives the same mechanical force to better mimic *in vivo* models. Colony organization of the 3D cell culture models is an important endpoint for phenotypic profiling, which can also be mapped to the genomic aberrations. Previous studies have shown that colony organization for HMEC can be round and lumen forming, non-lumen forming, flat, or flat and invasive^25^,^26^. The end result is a series of well-defined experiments that addresses the knowledge gaps between colony formation and increased stiffness, of the microenvironment, quantitatively, which is performed using confocal microscopy, advanced computational models^27^ (developed in our laboratory), and integrative morphometric and molecular profiling for hypothesis generation. Collectively, these analyses have revealed that ERBB2 is overexpressed, in a 3D cell culture model of MCF10A, when the stiffness of the microenvironment is at the same level of high mammographic density. In contrast, ERBB2 is not overexpressed in 184A1 HMEC cell line, which is considered to be more genetically stable.

## Results

### Stiffness of the microenvironment, within the range of mammographic density, is quantified in histology sections

**Figure 1:**
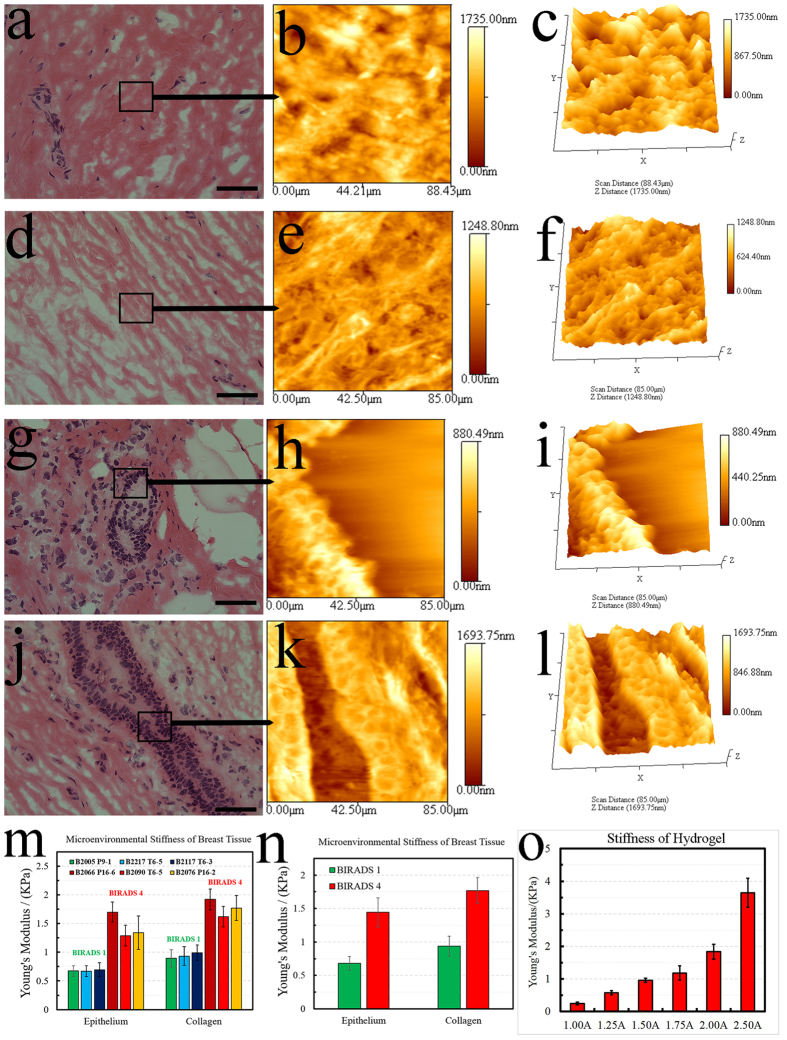
Topography and stiffness of the tissue compartments, from patients with low and high mammographic density (MD), are profiled with the Atomic Force Microscopy (AFM). The range of stiffness is then used to engineer matrices with similar characteristics. (**a**) A collagen region from low MD is selected from an H&E stained sample. (**b**) Selected region in (**a**) is profiled topographically by AFM. (**c**) Rendered 3D view of (**b**) showing the collagen topography from low MD. (**d**) A collagen region from high MD is selected from H&E stained sample. (**e**) Selected region in (**d**) is profiled topographically by AFM. (**f**) Rendered 3D view of (**d**) showing the collagen topography from high MD. (**g**) An epithelium region from low MD is selected from H&E stained sample. (**h**) Selected region in (**g**) is profiled topographically by AFM. (**i**) Rendered 3D view of the epithelium topography, in (**h**), from low MD. (**j**) An epithelium region from high MD is selected from H&E stained sample. (**k**) Selected region in (**j**) is profiled topographically by AFM. (**l**) Rendered 3D view of the epithelium topography, in (**k**), from high MD. (**m**) Stiffness of epithelium and collagen for low (BIRAD Score 1) and high MD (BIRAD Score 2) for each of the six patient. (**n**) Average value of the stiffness for each of the 3 patients in each subgroup. (**o**) Hydrogel-based matrices are modulated for their stiffness by varying concentration of agarose by 1%, 1.25%, 1.5%, 1.75%, 2.0% and 2.5%. These concentrations correspond to the mechanical stiffness of 250, 550, 950, 1200, 1800 and 3800 Pa, respectively. The first 5 stiffness values cover the range of MD. The scale bar for H&E stained images is 50 microns. The nomenclature of 1.00A, 1.25A, 1.50A, 1.75A, 2.00A and 2.50A represent concentration of agarose.

**Figure 2:**
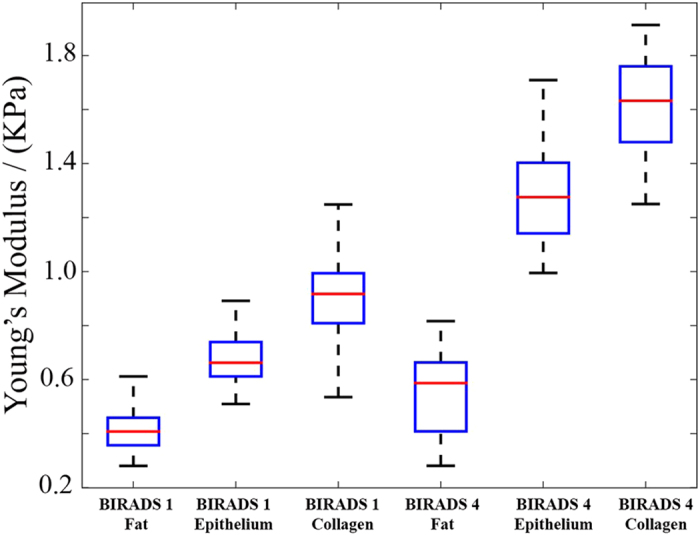
The distribution of indentation tests, profiled by AFM, is illustrated by box chart. The chart shows the range of stiffness for fat, epithelium and collagen regions for low and high mammographic density, respectively. The red bar denotes the median value of the associate box.

In order to establish the baseline stiffness for this study, AFM was used to characterize the matrix stiffness of breast tissue histology sections from patients with low and high MD who have gone through reduction mammoplasty. AFM provides an accurate spatial profiling of the stiffness of the microenvironment for each patch in a histology section. For each histology section, 3 patches were selected in triplicates (thus, a total of 9 patches) for collagen, epithelial, and fat regions. Each patch is 80-by-80 microns, and within each patch 9 grid points were randomly selected and nanoindented. The results are shown in Fig. 1, where, within small patches, H&E stained images and their corresponding AFM representations are shown for regions of low (Fig. 1a–c,g–i) and high (in Fig. 1d–f,j–l) MD. In addition, all indentation points are shown in Fig. 2, demonstrating the range distribution of stiffness for each category. The stiffness of the microenvironment for both collagen and epithelium were examined. The study:

- suggests that spatial organization of collagen has a preference for a phenotype with more fibrous in patients of high MD, as shown in Fig. 1e,f.
- establishes the baseline stiffness of ~800 and ~1800 Pa for the collagen regions, and ~500 and ~1100 Pa for epithelial in low and high MD, respectively, as shown in Fig. 1m,n.

In short, spatial organization of collagen appears less rough^28^ and fibrous in low MD. This qualitative observation complements previous observations that (i) fibrosis of collagen is associated with increased stiffness^29^,^30^,^31^, (ii) proportion of stroma is increased in tissue^32^,(iii) collagen from histology sections, of high mammographic density breast, is more stiffer as measured with atomic force microscopy^33^, and (iv) fibroblasts isolated from high MD accumulate less fat than fibroblasts from low MD under differentiation conditions, where fat accumulation leans toward reduced stiffness^34^. Furthermore, AFM profiling indicates that the epithelial regions are intrinsically stiffer in patients with high-MD, even though they are morphologically similar, as shown in Fig. 1h,i,k,l. This is an important observation, and remains an open problem whether epithelial cells are intrinsically different between low and high MD, or are simply responding to their microenvironment by reorganizing their cytoskeleton and subsequent changes in their genomic and epigenetic landscape.

### Concentration of agarose, in hydrogel, modulates stiffness of the microenvironment in 3D cell culture models

In order to mimic the stiffness of the microenvironment in low- and high-MD of breast tissues, agarose/Matrigel-based hydrogel is used to modulate the stiffness of the microenvironment in culture. Several strategies for modulating stiffness of the 3D of HMEC, using collagen and silk hydrogels, have been proposed in the past^35^,^36^. An initial pilot study, in our laboratory, led to the conclusion that a hydrogel-based substrate, containing different concentrations of agarose, can modulate stiffness more consistently. Agarose is non-biodegradable, and the literature supports that the stiffness of the proposed construct is maintained over extended culture^37^,^38^. More specifically, published literature has shown that stiffness of the microenvironment is maintained for over 30 days for chondrocytes and MSC in base media. It has also been demonstrated that normal murine mammary epithelial cells form intact 3D acini structures when grown within an agarose or Matrigel matrix^36^. With the hydrogel-based assay, stiffness can be easily modulated and calibrated with AFM. Figure 1o indicates modulation of the stiffness of the microenvironment from 250 to 4000 Pa by changing the concentration of agarose.

### 3D colonies, cultured in hydrogel, form polarized organization at low stiffness

**Figure 3:**
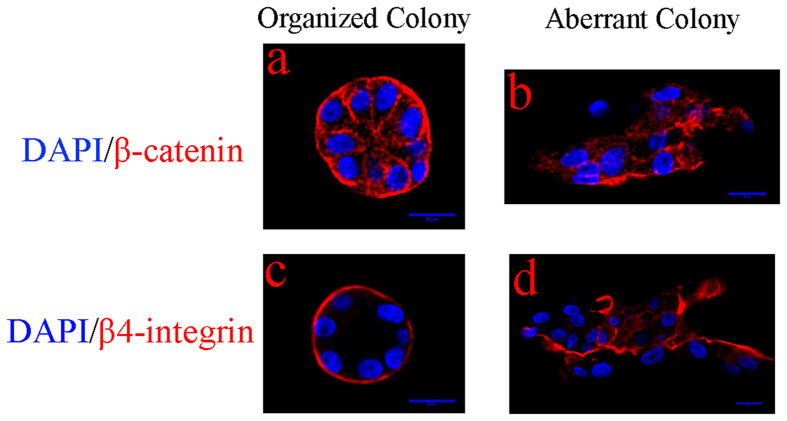
Immunofluorescent staining against β-catenin and β4-integrin is visualized with confocal microscopy. (**a**,**c**) Polarity is maintained at low stiffness. (**b**,**d**) Loss of polarity at high stiffness correlates with the aberrant colony organization. All colonies are stained with DAPI counterstain for visualizing nuclei.

To validate the hydrogel assay and to investigate changes in the colony polarity as a result of increased stiffness of the microenvironment, samples were labeled with a nuclear counterstain (*e.g*., DAPI), β-catenin, and β4-integrin, and were imaged with confocal microscopy as shown in Fig. 3. The results indicate that:

- At low stiffness, colony organization is homogeneous and remains polarized.
- At high stiffness, colony organization is heterogeneous, and in some cases, the loss of organization (*e.g*., hollow spherical architecture) is coupled with the loss of polarity, as shown in Fig. 3b,d.

### Colony organization is altered as a function increased stiffness

Colony organization, imaged with phase contrast microscopy, can partially aid in classifying genomic aberrations^26^,^27^. A more effective approach, however, requires nuclear counterstain, confocal microscopy of the entire sample volume, delineation of each nucleus in the colony from 3D images, and computing the indices that correspond to colony organization^27^. As a result, planar organization (as opposed to spherical ones), colony size, and loss of lumen formation can be computed more precisely. Such a phenotypic profiling enables global comparative analysis between experimental factors (*e.g*., different stiffness), heterogeneity analysis, and correlative analysis with the genome-wide molecular data for hypothesis generation. BioSig3D^39^ profiles a large number of indices associated with colony and nuclear indices, *viz*., nuclear shape and geometry, colony size, number of cells in a colony, flatness of colony, and a proxy for lumen formation. These indices are then analyzed for their mean and distribution properties, using BioSig3D.

### Mean-based analysis reveals correlation between increased stiffness and morphometric properties of colony formation

**Figure 4:**
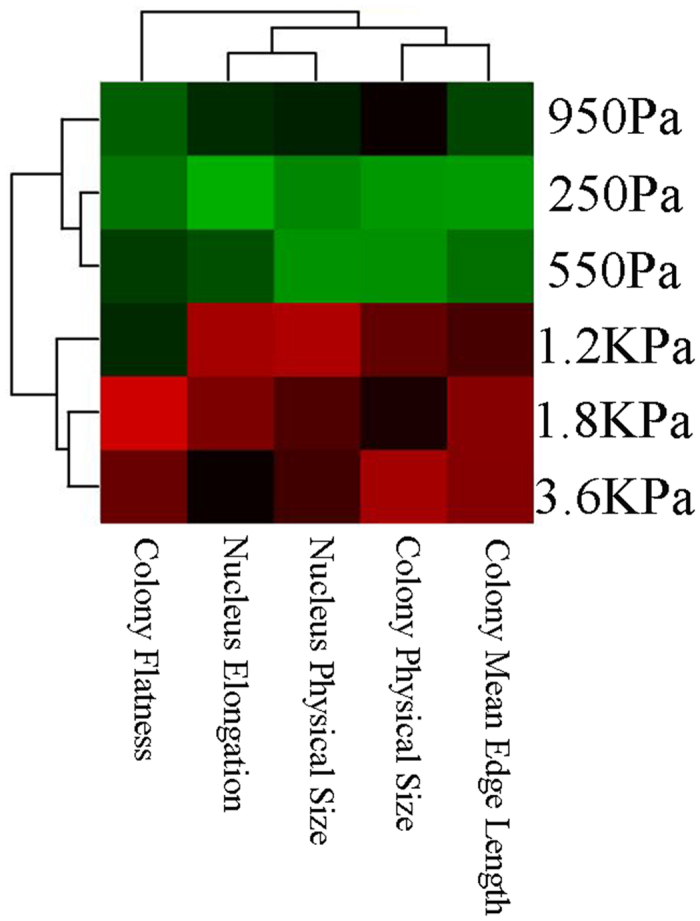
Heatmap clusters a subset of nuclear and colony organizational indices as a function of increased stiffness of the microenvironment. Global heatmap analysis indicates two intrinsic partitions.

Computed indices are normalized (*e.g*., zero mean with variance of one) independent of the experimental factor, and then visualized with a heatmap, with an example shown in Fig. 4. Red and green colors correspond to positive and negative values, respectively. Mean-based analysis reveals that increased stiffness:

-   partitions phenotypic signatures into two basic subgroups,
-   modulates colony organization in terms of flatness, size, and distances between adjacent nuclei in the same manner that genomically aberrant cell lines self-organize themselves^24^,^26^. Prior literature indicates that for a subset of genomically aberrant cell lines, such as triple-negative MDA-MB231 and EGFR amplified/ERBB2 MDA-MB468, colony flatness —increases, concurrent with the increased distances between adjacent cells^39^.
- correlates with the increased colony size and number of cells per colony, independent of the increased nuclear size. Increased nuclear size, as a function of stiffness, has been reported previously in 2D cultures^40^,^41^,^42^, but not in 3D assays. Therefore, increased nuclear size is independent of the 2D and 3D culture models.

However, a close observation indicates that these organization indices are not homogeneous, which reinforces the need for heterogeneity analysis, and for a more sensitive computational assay for comparative analysis.

### Colony formation is heterogeneous

**Figure 5:**
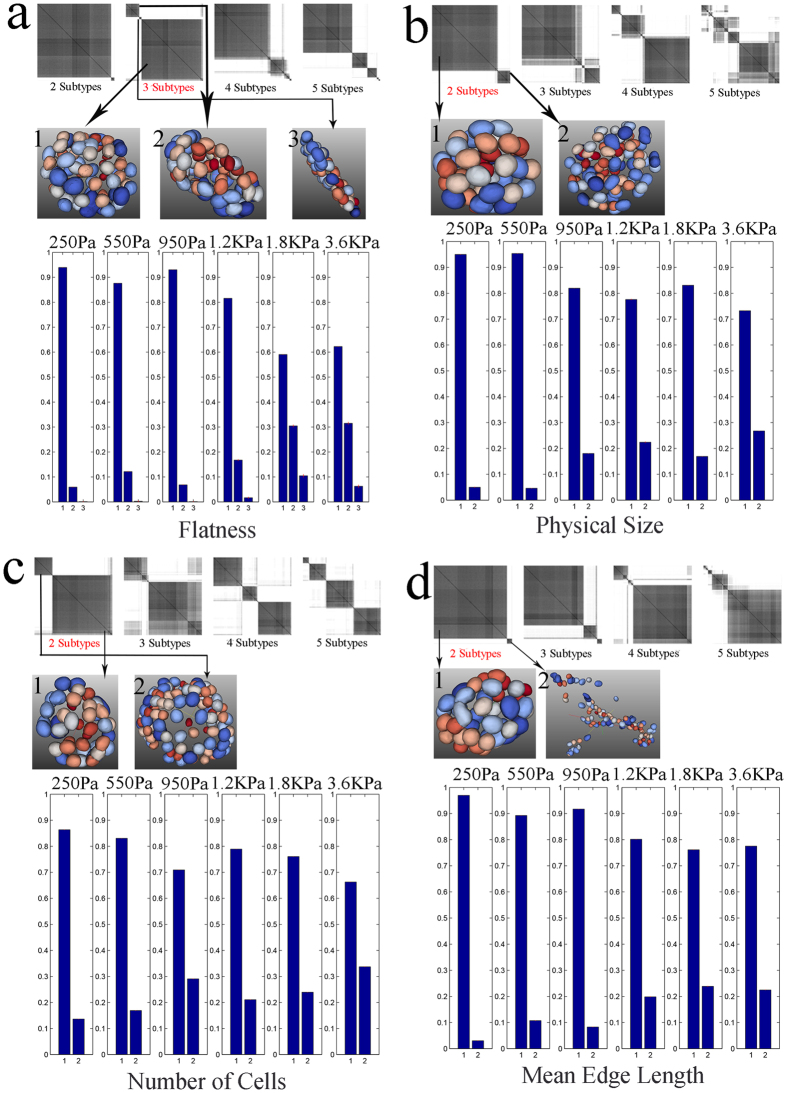
Heterogeneity analysis, for MCF10A, through consensus clustering, identifies subtypes for a number of computed morphometric indices as a function of (**a**) colony flatness, (**b**) colony physical size, (**c**) number of nuclei per colony, and (**d**) mean edge length between adjacent nuclei within a colony. The representative phenotype of the 3D colony is shown for each subtype by segmenting and rendering nuclei in the colony.

**Figure 6:**
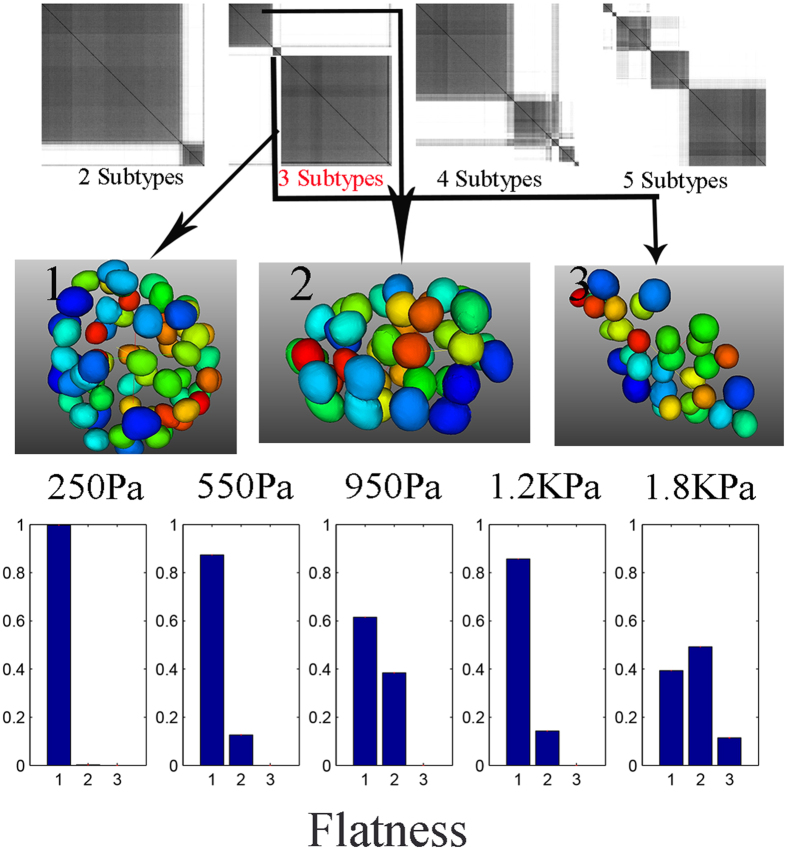
Heterogeneity analysis, for 184A1, through consensus clustering, identifies subtypes for colony flatness. The representative phenotype of the 3D colony is shown for each subtype by segmenting and rendering nuclei in the colony.

Heterogeneity is usually computed in terms of entropy^43^,^44^ or higher-order statistics, which is adequate for having an index for describing distribution of computed indices. A more powerful approach is to apply consensus clustering^45^ and to identify subtypes within the population. Subtyping and mean-based analysis can be used together synergistically. Subtyping is an interactive module, which has been implemented and validated in BioSig3D. First, consensus clustering is applied to a specific computed index in a dataset, regardless of the experiment factors, where a single representative sample in each subtype can be queried and visualized. Second, within each experimental set of experimental factors, frequency of occurrence of each subtype is computed and represented as a bar chart. This protocol has been applied to compute heterogeneity as a function of colony size, number of cells per colony, flatness of colonies, and a proxy for lumen formation index. Figure 5 shows the outcome of heterogeneity analysis for MCF10A, where computed indices are clearly modulated as a function of stiffness. Four observations are made about this increased stiffness. First, the nuclear size is increased. Second, the physical sizes of the colonies are also increasing, and because the number of cells in a colony is also increasing (*e.g*., increased proliferation), then the colony sizes are increasing independent of the nuclear size. Third, the frequency of aberrant flat colonies is increasing. Fourth, the distances between adjacent cells, within a colony, are also increasing. These observations are concomitant with aberrant colony organizations, per previous literature^46^,^47^. The above observations (i) correlate with mean-based analysis, (ii) demonstrate the morphological trends as a function of increased stiffness, and (iii) identify the intrinsic phenotype of each subtype that might otherwise be hidden. In addition, heterogeneity analysis for HMEC 184A1 indicates that the frequency of aberrant colony formation is increased as a function of higher stiffness of the microenvironment, as shown in Fig. 6. This observation is consistent with the MCF10A cell lines.

### Colony heterogeneity remains persistent over time

In order to verify that heterogeneity is persistent over an extended culture period, an experiment was designed to quantify colony formations on days 9 and 21. The result is shown in Supplementary Figure 1, which indicates that the percentage of the round colonies remains at 80% and 50% for low and high stiffness, respectively, independent of the harvest time.

### Genome-wide transcriptome profiling hypothesizes common regulators

To investigate changes in gene expression as a result of increased stiffness, genome-wide profiling was performed, in parallel, with phenotypic profiling, i.e., exact same culture conditions. Expression data were analyzed using several complementary methods.

**Figure 7:**
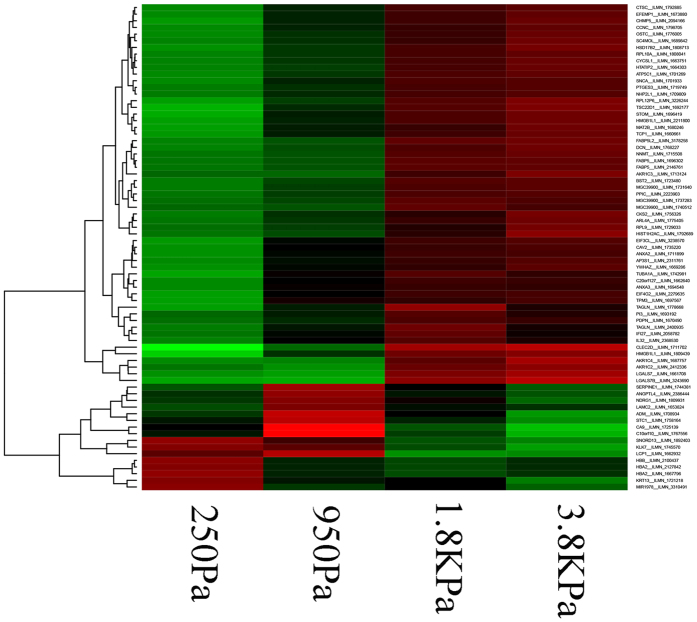
Transcriptome analysis shows a subset of genes that are up/down-regulated as a function of increased stiffness of the microenvironment. The experimental conditions (e.g., stiffness) covers the range of mammographic density.

- Figure 7 and Supplementary Table 1 show 133 genes with a p-value of <0.001 that are modulated with increased stiffness, where there is a clear transition pattern of up/down regulation for a specific set of genes. These 133 genes were further enriched for bioinformatics analysis. Utilizing the Panther classification system, bioinformatics analysis identified enrichment of the structural molecular activities (*e.g*., ECM constituent, structural constituent of the cytoskeleton), with a p-value of 0.017. There were no other meaningful enrichments. Enrichment of structural activities is expected as cellular morphology and colony organization are altered as a result of increased stiffness. Furthermore, transcripts were evaluated for their dependencies with breast cancer biomarkers, and in particular ERBB2:
  - TUBA1A, involved in G2/M transition, has been shown to be a part of a network that is associated with ERBB2 positive tumors in clinical samples^48^;
  - Annexin A2 (ANXA2) has been found to be inversely correlated with ERBB2 expression in clinical samples and breast cancer cell lines^49^; and
  - TAGLN is activated by ERBB2 signaling by way of the Ingenuity Pathway Analysis (IPA).
- Utilizing IPA core analysis for the top 133 differentially expressed transcripts, two sets of analysis were performed:
  - Network analysis partitioned these genes into 4 distinct functional categories, with Networks shown in Supplementary Table 2 and Supplementary Figure 2a to Supplementary Figure 2d.
  - Upstream analysis revealed a list of regulators, including ERBB2, with a p-value of 0.016 as shown in Supplementary Figure 3.

Finally, genome-wide expression data were correlated with computed phenotypic indices such as colony size and flatness. In the case of colony size, correlation coefficients of 0.9 or better (in both directions), and with the p-value of 0.02 or better, were filtered for further enrichment analysis. ERBB2 was identified as an upstream regulator, with a p-value of 0.006. Similar analysis with colony flatness revealed PPARG as an upstream regulator. These analyses hypothesize ERBB2 as a potential endpoint for validation, even though differential gene expression analysis does not show ERBB2 as having a strong p-value.

### Stiffness of the environment exacerbates the premalignant status of MCF10A

**Figure 8:**
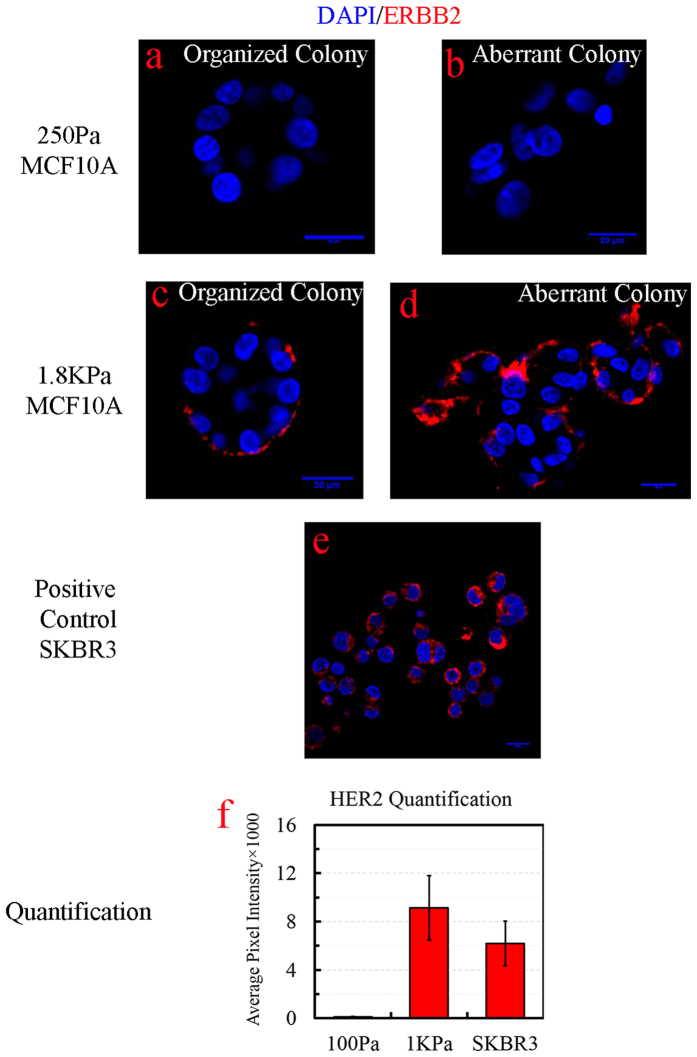
ERBB2 overexpression is imaged, by fluorescent microscopy, in a culture condition that mimic stiffness of the high MD for MCF10A. (**a**,**b**) ERBB2 is not expressed at low stiffness of 250 Pa independent of colony organization. (**c**,**d**) ERBB2 is overexpressed at high stiffness of 1800 Pa independent of the colony organization. (**e**) Malignant line of SKBR3 overexpresses ERBB2 in 3D (positive control). (**f**) Quantitative analysis of ERBB2 expression for low and high stiffness with positive control as reference. The scale bar represents 20 μm.

Overexpression of ERBB2 is associated with aberrant colony formation in culture (*e.g*., in SKBR3 cell lines) and in approximately 20% of the patients (*e.g*., Luminal B with ER^+^ and/or PR^+^ with HER2^+^, ER^−^ & PR^−^ &HER2^+^). Because genome-wide transcriptome analysis has also enriched ERBB2 signaling, samples have been stained with ERBB2 antibodies in each of the treatment groups. Results indicate that ERBB2 is overexpressed at high stiffness values of the microenvironment that correspond to high MD. A representative example is shown in Fig. 8, where ERBB2 is consistently overexpressed, independent of the colony shape, as a result of high stiffness of the microenvironment. However, overexpression of the ERBB2 protein, observed by immunofluorescence microscopy, does not correlate directly with its gene expression. The correlations between mRNA and protein abundance can be very low in biological samples^50^,^51^, which is potentially due to a number of variables, including differential rates of transcription and translation between mRNA and protein expression, and a differential half-life between mRNA and protein.

To investigate if ERBB2 overexpression is intrinsic to high stiffness for 3D cell culture models, MCF10A were also cultured in 2D in the presence and absence of Matrigel with the stiffness values in the range of low and high MD. This experiment concluded that ERBB2 is not overexpressed in monolayers. However, overexpression of ERBB2 in 3D cell culture models, within the range of MD, raises another question on how ubiquitous this observation will be. Hence, another HMEC with a more genomically stable characteristic, 184A1, was cultured in 3D under similar stiffness conditions. 184A1 did not overexpress ERBB2 at high stiffness; thus, it must be that the pre-malignant cell line, MCF10A, is more sensitive to the increased stiffness of the microenvironment.

## Discussions

Matrix stiffness plays an important role in the cellular growth, differentiation, morphology, and motility, and studies have shown that normal breast tissue is soft and of the order of 200 Pascal (Pa), whereas tumor tissue is quite stiff and of the order of 4000 Pa. Stiffness of the microenvironment has been measured in normal and tumor regions of mammary glands in mice^5^ and in human^52^, and in both species, tumor regions are consistently stiffer, of the order of 4000 Pa. However, the link between MD and stiffness of the microenvironment are established, and is presented here from samples with a known MD that were collected through reduction mammoplasty. Subsequently, stiffness of the microenvironment was modulated by varying the agarose concentration in hydrogel within the range of the MD. One of the main barriers in phenotypic profiling of 3D colonies is heterogeneity. Colony heterogeneity originates from technical and biological variations. The most important component of the technical variation has to do with the culture method, which can utilize either the “ontop” or “embedded” protocol^24^. Compared to the “ontop” protocol, “embedded” cultures are more labor-intensive, and require additional scaffolding, but tend to be more clonal if the initial seeding is sparse. Furthermore, each cell in the colony is exposed to a similar level of stiffness, and, as a result, any technical variation resulting from the cells in the same colony receiving variable force is reduced.

Having reduced the technical variations, 3D colony profiling revealed that aberrant colony formation is gradually increased as a function of the stiffness of the microenvironment. These observations are consistent for MCF10A and 184A1. Quantitative profiling was performed using mean-based analysis and subtyping, to investigate global changes as well as heterogeneity in colony formation. In short, stiffness modulates aberrant colony formation. Parallel cultures for genome-wide transcriptome profiling hypothesized ERBB2 as a potential regulator in MCF10A, which was subsequently validated in 3D cultures at astiffness value corresponding to the high MD. However, ERBB2 was not overexpressed in (i) a genomically stable cell line of 184A1, and (ii) in 2D cultures of MCF10A at the high stiffness value of the microenvironment. Therefore, overexpression of ERBB2 is strictly a 3D effect and is exacerbated by the pre-malignant state of the MCF10A cell line.

In summary, it is concluded that stiffness, within the range of MD, plays a major role in promoting aberrant colony organization with clinically relevant upregulation of ERBB2 in MCF10A. Because women with high MD have an increased risk factor to develop breast cancer, it is necessary to investigate the overexpression of ERBB2 at high stiffness further. A second experiment with a genomically stable 184A1 HMEC revealed that ERBB2 is not overexpressed with the increased stiffness. Although both MCF10A and 184A1 are immortalized premalignant cell lines, MCF10A has probably lost more barriers toward transformation, and uniquely expresses ERRB2 under high stiffness of the microenvironment. In this particular case, stiffness exacerbates the premalignant phenotype.

## Materials and Methods

### Acquisition of histology sections from low and high MD

Normal breast histology sections from low and high MD (BIRADS score 1 and 4) were collected from patients who had undergone reduction mammoplasty at UCSF. For each BIRADS score, tissue sections from 3 different patients were collected, and, for each patient, 4 non-stained and 2 H&E stained sections were acquired at 10 micron thickness. Tissue sections were collected and transported in frozen conditions. Landmarks associated with distinct regions of microanatomy (*e.g*., collagen, epithelial, fat) were identified and marked in the H&E stained images, and subsequently matched in adjacent non-stained sections, for profiling with atomic force microscopy (AFM).

### 2D culture model

The 2D culture model utilizes polyacrylamide (PA) hydrogel, which is modulated between 480 Pa to 4470 Pa. The details of the PA hydrogel preparation is outlined in Lin *et al*.^53^. After the gels were solidified, the gels were rinsed with 70% ethanol for 30 mins in order to sterilize. PBS solution was then used to wash the gels. Matrigel (BD 354230, LOT36147) was diluted with 1:10 ratio, added to cover the surface of the gel, and incubated for 1 h in ambient conditions. After carefully removing the excess Matrigel solution, 8000 MCF10A cells were seeded on the PA hydrogel, and incubated for 24 h for cell attachment. Next, the hydrogels with cells attached were incubated for 8 days while changing the medium once every third day.

### Embedded 3D culture model

The 3D cell culture model utilizes embedded protocol as opposed to the “ontop” method^24^. For each stiffness condition, 3D cell cultures were replicated three times with MCF10A and 184A1 HMEC and stiffness of the microenvironment was modulated by varying concentration of agarose. The initial seeding density, for both cell lines, is at 8000 cells per well, and cells were carefully suspended in 40 μL Matrigel matrices. Next, 40 μL of 2.0%, 2.5%, 3%, 3.5%, 4% and 5% agarose solution (Sigma) were added and thoroughly mixed. The final concentrations were at 1.0%, 1.25%, 1.5%, 1.75%, 2.0% and 2.5% of agarose gels supplemented with Matrigel matrices (BD 354230, LOT36147), which correspond to stiffness of 250, 550, 950, 1200, 1800, and 3600 Pa, respectively. The mixtures were placed in an 8-well chambered coverglass (Nunc Lab Tek II), and incubated for 20 min, allowing each matrix to gel before adding the medium (500 μL/well). Cells were maintained in culture for 8 days while changing the medium once every third day. Changing of medium was also monitored by phase microscopy for proper growth and colony formation. The experiment was repeated in triplicates, and each gel contains 50–100 colonies.

### Mechanical characterizations by Atomic Force Microscopy (AFM)

AFM was used to quantify the local stiffness within the histology sections as well as engineering matrices for modulating local tension. Briefly, AFM indentation experiments were carried out with a MFP-3D AFM (Asylum Research, Santa Barbara, CA, USA). A special AFM tip, fitted with a borosilicate glass sphere (5 μm in diameter, 0.05 N × m^−1^ of spring constant), was acquired from Novascan Tech. The AFM tip was brought down, with maximum strain set at 20% to indent the samples. Next, the Young’s Modulus was calculated. Tissue slides (3 frozen and 1 HE stained) from 3 patients for each category of mammographic density were used for the AFM profiling. H&E stained slide was used to select three regions per tissue compartment (e.g., epithelium, fat, collagen), which adds up to 9 regions per tissue section and 54 regions for the entire sample size. For each region, specified on the H&E stained slide, the corresponding region on the frozen samples was marked. Frozen samples were then thawed, and the size of each region was set at 80 μm × 80 μm. Each region was divided into 9 sub-regions for individual spot test. 3 spot measurements (e.g., triplet indentation), in each sub-region, were performed. These data were then plotted as the mean ± standard deviation (SD) in Fig. 1. The population profiling, for 81 point, is included in the supplementary Fig. 4 as a box plot. The same protocol was applied to measure spatial stiffness of histology sections as well as for calibrating stiffness of the engineered matrices for the embedded 3D cell culture model.

### Cell subculture and maintenance

Two HMEC were cultured sequentially following an initial pilot project, validation, and hypothesis generation. These were MCF10A and 184A1. The MCF10A model system was obtained from ATCC at passage >50, and was then subcultured (P5-P7) for the proposed studies. The 184A1 cells were obtained from the Martha Stampfer lab^54^, and passages 53–60 were utilized. Cells were maintained in a 37 °C incubator with 5% CO_2_. MCF10A were cultured in DMEM/F12 medium (Life Science Technology) supplemented with 5% horse serum (Life Science Technology), 10 ng/mL FGF (Life Science Technology), 50 ng/mL chlora toxin (Sigma), 200 μg/mL insulin (Sigma), 100 ng/mL hydroxylcortisone (Sigma), and 1% penicillin-streptomycin (Life Science Technology). 184A1 cells were cultured in M87A medium, which was provided by Martha Stampfer’s lab at the Lawrence Berkeley National Lab.

### Fixation and immunofluorescence staining

Cells in the hydrogel were fixed at room temperature in 10% formalin for 30 min. After 3 times of PBS washes, cells were permeabilized using a 0.1% Triton X-100 solution for 5 min. Hydrogels were washed 3 times with PBS, blocked using a 1% bovine serum albumin (BSA) solution (Sigma) supplemented with 1% goat IgG (Jackson ImmunoResearch), and the blocking solution was maintained for 1 h. In some cases, samples were stained for a specific molecular endpoint, but in all cases, DAPI nuclear stain was applied for profiling colony organization. Monocolonal rabbit anti human primary antibodies (*e.g*., ERBB2, β-catenin, β4-integrin) (Abcam) were diluted in blocking solution (1:500), incubated for 1 h, and followed by 3 PBS washes. Goat anti rabbit secondary antibody conjugated with Alexa 488 and Alexa 565 (Life Science Technology) was diluted in the blocking solution (1:250), conjugated to primary antibody for 1 h, and followed by 3 PBS washes. Subsequently, nuclei were stained with 4′-6-diamidino-2-phenylindole (50 ng/mL) (DAPI, Life Science Technology).

### Fluorescent Microscopy

Stained samples were imaged using a Zeiss LSM 710 system equipped with a Zeiss Apochromat 40X/1.1 (0.8 mm WD) water immersion objective lens. The excitation filters were set at 405 and 561 nm, and the emission filters were set to receive signals between 420–480 and 597–700 nm, respectively. The laser intensity was set at 1%. A twin-gate main beam splitter with two wheels, each wheel having 10 filter positions (and thus 100 possible combinations), was used to separate excitation and emission beams. The pinhole was set at “1”. The digital gain was adjusted to approximately ¾ of the maximum gain, which kept the dynamic range of the pixels between 500–2000 (12 bits). The 3D stack function of the Zen software was used to collect raw 3D information for each colony, and was concurrent with “Z correction” of the pixel intensity for thick samples. The voxel size was set to 0.25 μm × 0.25 μm × 1 μm, the image files were saved as lsm files, and uploaded to the BioSig3D imaging bioinformatics system^39^.

### RNA analysis

Total RNA was extracted using a Qiagen RNAeasy micro kit, following the in-kit protocol. The RNA quality was checked to assure that RNA integrity number (RIN) was over 7, out of maximum 10. Total RNA extractions were sent to the UCLA UNGC core facility for profiling, where UCLA performed their own quality control check independently. Subsequently, UCLA amplified the total RNA by Ambion amplification and profiled gene expression on Illumina human ht-12 chip in triplicates. The chips were scanned, gene expression data were normalized, and p-values for the quality of each gene was computed, and a spreadsheet was delivered for integrative analysis.

### High-content profiling of colony organization in 3D cell culture models

3D Colony organizations were computed by segmenting nuclear regions, from 3D images, and by computing a series of geometric and graphical features per colony. Geometrical features (*e.g*., colony flatness) represent colony shape attributes globally. Graphical features (*e.g*., lumen formation index) represent local attributes of the colony organization. These analyses were performed by BioSig3D, which was also developed by our Laboratory. This is publicly available at biosig.lbl.gov, and a special version of it will be released as a virtual machine with the data that have been collected for this manuscript. BioSig3D provides an end-to-end solution for designing high-content screening assays, robust algorithms for quantitative analysis of multicellular systems and their organization, visualization of raw and processed 3D volumetric data, and integration of bioinformatics analysis. As a result, colony organization can be represented as a heat map or similarity matrices that correspond to colony heterogeneity. In this paper, BioSig3D has been utilized to characterize heterogeneity in colony formation for cell lines. For each experimental condition, 40 3D datasets were uploaded for analysis.

### Ethic statement

Written form consent was obtained from all subjects. The procedure to collect and use human tissue was carried out in accordance with approved guideline. All methods were approved by University of California San Francisco, Lawrence Berkeley National Lab and University of Nevada Reno.

## Additional Information

**How to cite this article**: Cheng, Q. *et al*. Stiffness of the microenvironment upregulates ERBB2 expression in 3D cultures of MCF10A within the range of mammographic density. *Sci. Rep*. **6**, 28987; doi: 10.1038/srep28987 (2016).

## Supplementary Material

Supplementary Information
