# Erratum: Stiffness of the microenvironment upregulates ERBB2 expression in 3D cultures of MCF10A within the range of mammographic density

**DOI:** 10.1038/srep31680

**Published:** 2016-08-24

**Authors:** Qingsu Cheng, Cemal Cagatay Bilgin, Gerald Fontenay, Hang Chang, Matthew Henderson, Ju Han, Bahram Parvin

Scientific Reports 6: Article number: 2898710.1038/srep28987; published online: 07 07 2016; updated: 08 24 2016

The original version of this Article contained a typographical error in the spelling of the author Gerald Fontenay, which was incorrectly given as Gerald Fonteney. This has now been corrected in the PDF and HTML versions of the Article.

