# Supplementary material for: Stiffness of the microenvironment upregulates ERBB2 expression in 3D cultures of MCF10A within the range of mammographic density
Source: Sci Rep. 2016 Jul 7;6:28987. doi: 10.1038/srep28987 (PMC4935956; doi:10.1038/srep28987)
Supplement: Supplementary Information [file srep28987-s1.pdf]

Supporting information

Stiffness of the microenvironment upregulates ERBB2 expression in 3D cultures of MCF10A within the range of mammographic density

Qingsu Cheng [1,2], Cemal Cagatay Bilgin[1], Gerald Fonteney[2], Ju Han[1,2], Matthew Henderson[1], Hang Chang[1,2,] Bahram Parvin[1,2,]*

1 1664 N Virginia Street, Department of Electrical and Biomedical Engineering, University of Nevada, Reno, Reno NV, 89557, USA

2 Lawrence Berkeley National Laboratory, 1 Cyclotron Road, MS-977, Life Sciences Division, Berkeley CA, 94720, USA.

* Corresponding author:
Bahram Parvin
E-mail: bparvin@unr.edu

Supplementary Table 1. Genes in Figure 4 are listed from top to the bottom.

CTSC__ILMN_1792885
EFEMP1__ILMN_1673880
CHMP5__ILMN_2094166
CCNC__ILMN_1798705
OSTC__ILMN_1776005
SC4MOL__ILMN_1689842
HSD17B2__ILMN_1808713
RPL10A__ILMN_1808041
CYCSL1__ILMN_1663751
HTATIP2__ILMN_1664303
ATP5C1__ILMN_1701269
SNCA__ILMN_1701933
PTGES3__ILMN_1719749
NHP2L1__ILMN_1709809
RPL12P6__ILMN_3226244
TSC22D1__ILMN_1692177
STOM__ILMN_1696419
HMGB1L1__ILMN_2211800
MAT2B__ILMN_1680246
TCP1__ILMN_1660661
FABP5L2__ILMN_3178258
DCN__ILMN_1768227
NNMT__ILMN_1715508
FABP5__ILMN_1696302
FABP5__ILMN_2146761
AKR1C3__ILMN_1713124
BST2__ILMN_1723480
MGC39900__ILMN_1731640
PPIC__ILMN_2223903
MGC39900__ILMN_1737283
MGC39900__ILMN_1740512
CKS2__ILMN_1756326
ARL4A__ILMN_1775405
RPL9__ILMN_1729033
HIST1H2AC__ILMN_1792689
EIF3CL__ILMN_3238570
CAV2__ILMN_1735220
ANXA2__ILMN_1711899
AP3S1__ILMN_2311761
YWHAZ__ILMN_1669286
TUBA1A__ILMN_1742981
C20orf127__ILMN_1662640
ANXA3__ILMN_1694548
EIF4G2__ILMN_2279635
TPM3__ILMN_1697567
TAGLN__ILMN_1778668
PI3__ILMN_1693192
PDPN__ILMN_1670490
TAGLN__ILMN_2400935

IFI27__ILMN_2058782
IL32__ILMN_2368530
CLEC2D__ILMN_1711702
HMGB1L1__ILMN_1809439
AKR1C4__ILMN_1687757
AKR1C2__ILMN_2412336
LGALS7__ILMN_1661708
LGALS7B__ILMN_3243690
SERPINE1__ILMN_1744381
ANGPTL4__ILMN_2386444
NDRG1__ILMN_1809931
LAMC2__ILMN_1653824
ADM__ILMN_1708934
STC1__ILMN_1758164
CA9__ILMN_1725139
C10orf10__ILMN_1767556
SNORD13__ILMN_1892403
KLK7__ILMN_1745570
LCP1__ILMN_1662932
HBB__ILMN_2100437
HBA2__ILMN_2127842
HBA2__ILMN_1667796
KRT13__ILMN_1721218
MIR1978__ILMN_3310491

Supplementary Table 2. Network analysis partitioned the top 133 genes, in Figure 4, into four distinct functional categories.

ID	Molecules in Network	Score	Focus Molecules	Top Diseases and Functions
1	ACTB, Alpha tubulin,ANXA2,ATP5C1,Beta Tubulin,CCNC,CFL1,ERBB2,FABP5,FOXA1,HBA1/HBA2, HBB,HES1,Histone h3,Histone-h4,Hsp70,Hsp90,HTATIP2,JAK1,KRT13,MMP25,MYOCD,NCF1,PTGES3,RNA polymerase II,RPL10A,RUNX2,SNCA,STOM,TAGLN,TCP1,TINF2,TPM3,TUBA1A,YWHAZ	38	17	Free Radical Scavenging, Cell Death and Survival, Connective Tissue Disorders
2	ANXA3,AP3S1,ARL4A,BAG3,BCL2,CHMP5,CKS2,CLOCK,CTSC,EIF3CL,EIF4G2,HECW2,HIST1H2AC,HMGA1,INSIG2,ISG15,KCNMA1,KLK7,LGALS7/LGALS7B,LOC728026,MAT2B,MSMO1,MYCN,NNMT,PPIC,SMAD3,SP3,TCF3,TP63,TUBG1,UBC,USP1,USP15,USP38,YY1	36	16	Cancer, Organismal Injury and Abnormalities, Skeletal and Muscular Disorders
3	AGR2,BCL2,BST2,CEBPA,DCN,EFEMP1,ERBB2,FOXA2,FOXP3,HDAC2,HES1,IFI27,IL32,KLF4,KLF5,LCP1,MTPN,NR3C1,OSTC,PDPN,PI3,PIAS1,POU5F1,RELA,RUNX2,SMAD7,SMARCA2,SOX11,SRF,TAGLN,TGFB1,TMSB15B,TP63,TSC22D1,YAP1	25	12	Gene Expression, Cellular Development, Tissue Development
4	20α-hydroxysteroid dehydrogenase,3(or 17)α-hydroxysteroid dehydrogenase,3α-hydroxysteroid dehydrogenase (A-specific),ACD,ACTB,AKR1C3,AKR1C4,AKR1C1/AKR1C2,APBB1,CAV2,ERBB2,ESR1,FHL2,FOXA1,GATA4,HNF4A,HOXC8,HSD17B2,KLF5,LGALS7/LGALS7B,MKL1,NADH or NADPH:quinone oxidoreductase,NHP2L1,NOTCH3,NUFIP1,RNA polymerase I,RNA28S5,RPL9,S100A9,SMARCA2,SNORD13,TINF2,trans-1,2-dihydrobenzene-1,2-diol dehydrogenase,UBC,USP15	20	10	Endocrine System Development and Function, Energy Production, Small Molecule Biochemistry

Figure captions

Supplementary Figure 1. Heterogeneity in colony formation between days 9 and 21 remains stable. Heterogeneity is measured by percentages of colonies being classified as round versus flat.
Supplementary Figure 2. Network analyses, using IPA, for each of the four functional groups in Supplementary Table 2 are shown. (a)Network involved in free radical scavenging, cell death and survival, and connective tissue disorders. (b) Network involved in cancer, organismal injury and abnormalities, and skeletal and muscular disorders. (c) Network involved in cellular development and tissue development. (d) Network involved in endocrine system development and function, energy production, and small molecule biochemistry.
Supplementary Figure 3. Upstream analysis, using IPA, reveals ERBB2 as one of the hubs.

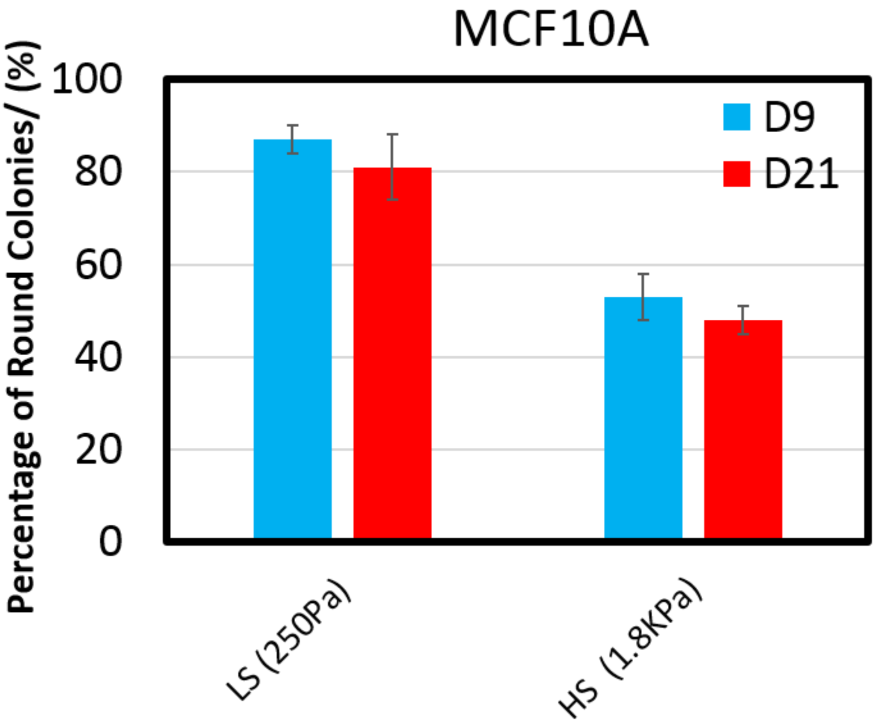

Supplementary Figure 1. Heterogeneity in colony formation between days 9 and 21 remains stable. Heterogeneity is measured by percentages of colonies being classified as round versus flat.

a

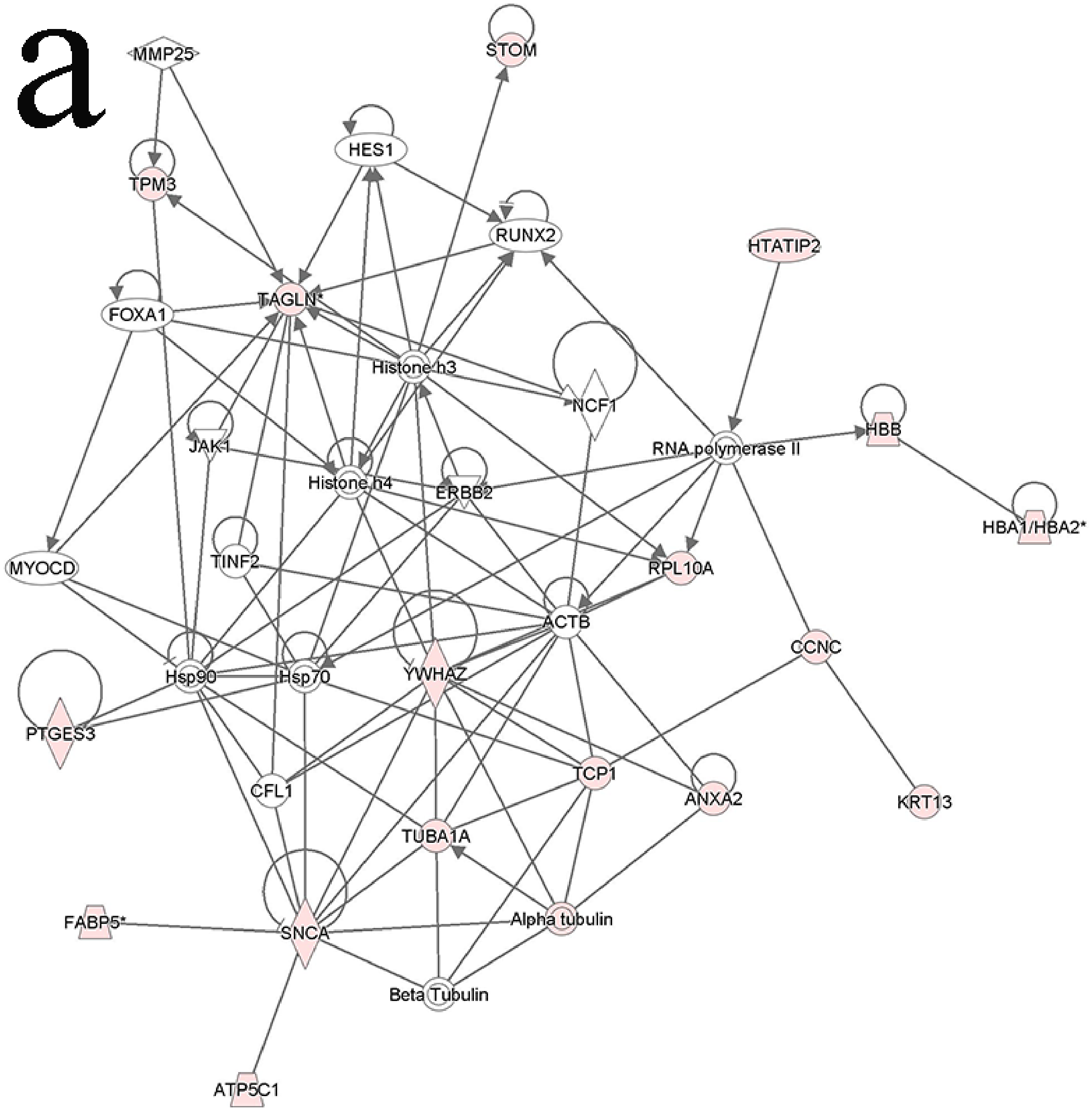
MMP25
STOM
HES1
TPM3
RUNX2
HTATIP2
FOXA1
TAGLN*
Histone h3
NCF1
JAK1
HBB
RNA polymerase II
Histone h4
ERBB2
HBA1/HBA2*
TINF2
MYOCD
RPL10A
ACTB
CCNC
Hsp90
Hsp70
YWHAZ
PTGES3
CFL1
TCP1
ANXA2
KRT13
TUBA1A
FABP5*
SNCA
Alpha tubulin
Beta Tubulin
ATP5C1

b

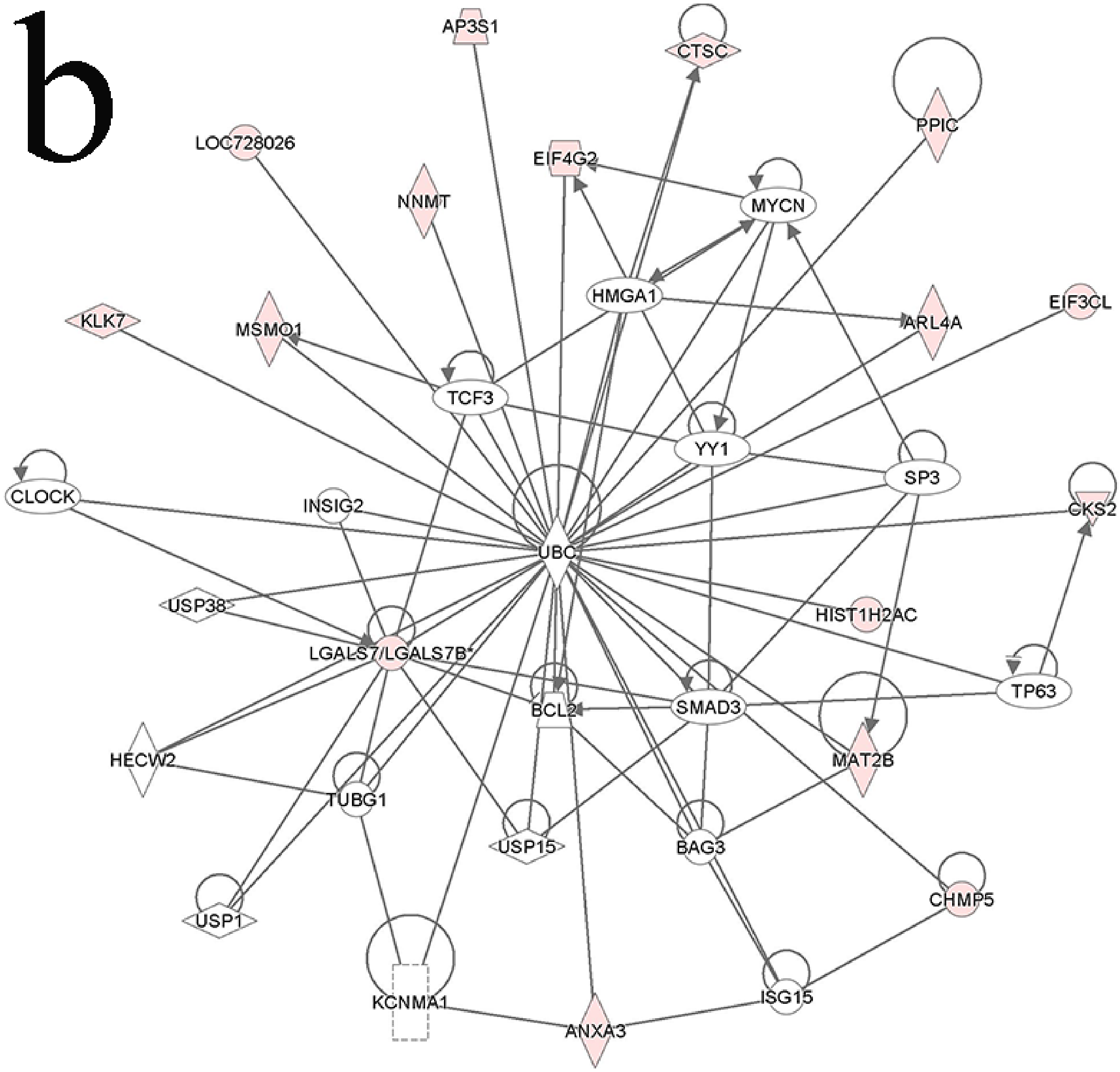
AP3S1
CTSC
PPIC
LOC728026
EIF4G2
NNMT
MYCN
HMGA1
EIF3CL
KLK7
MSMO1
ARL4A
TCF3
YY1
SP3
CLOCK
INSIG2
CKS2
UBC
USP38
HIST1H2AC
LGALS7/LGALS7B*
TP63
BCL2
SMAD3
HECW2
MAT2B
TUBG1
USP15
BAG3
CHMP5
USP1
ISG15
KCNMA1
ANXA3

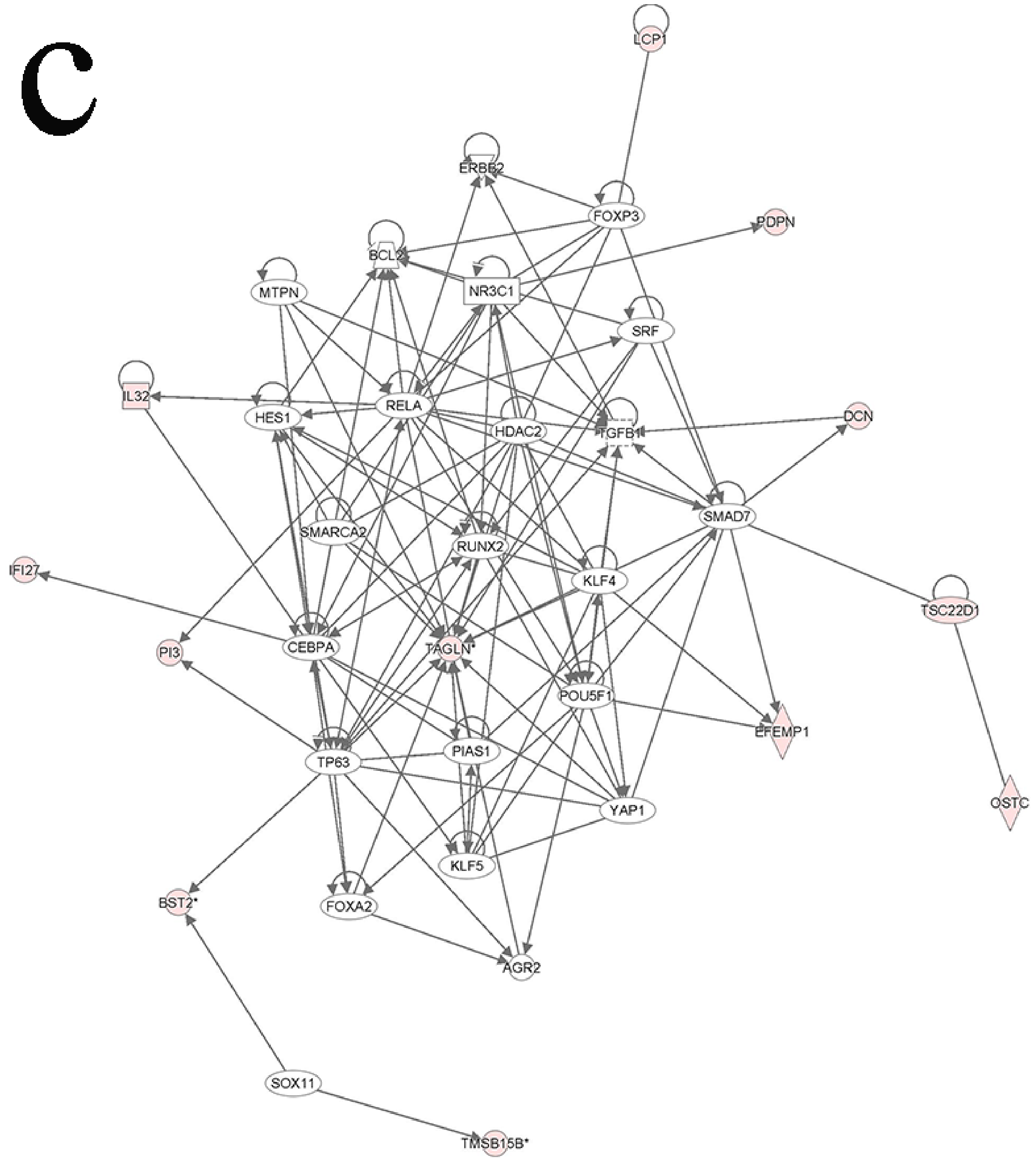
C
LCP1
ERBB2
FOXP3
PDPN
BCL2
NR3C1
MTPN
SRF
IL32
RELA
HES1
HDAC2
TGFB1
DCN
SMAD7
SMARCA2
RUNX2
KLF4
IFI27
TSC22D1
PI3
CEBPA
TAGLN*
POU5F1
EFEMP1
TP63
PIAS1
YAP1
OSTC
KLF5
BST2*
FOXA2
AGR2
SOX11
TMSB15B*

d

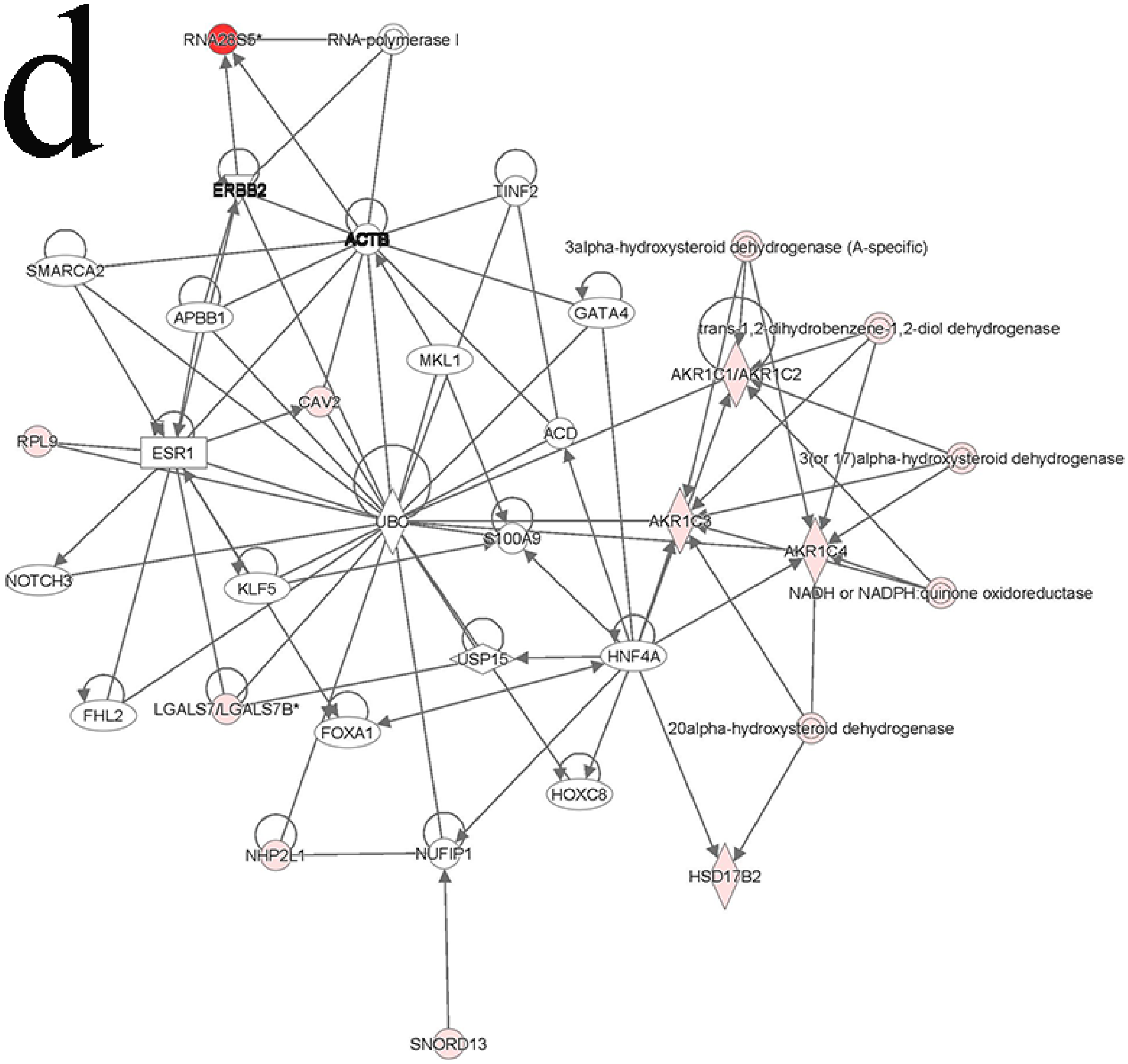

Supplementary Figure 2. Network analyses, using IPA, for each of the four functional groups in Supplementary Table 2 are shown. (a)Network involved in free radical scavenging, cell death and survival, and connective tissue disorders. (b) Network involved in cancer, organismal injury and abnormalities, and skeletal and muscular disorders. (c) Network involved in cellular development and tissue development. (d) Network involved in endocrine system development and function, energy production, and small molecule biochemistry.

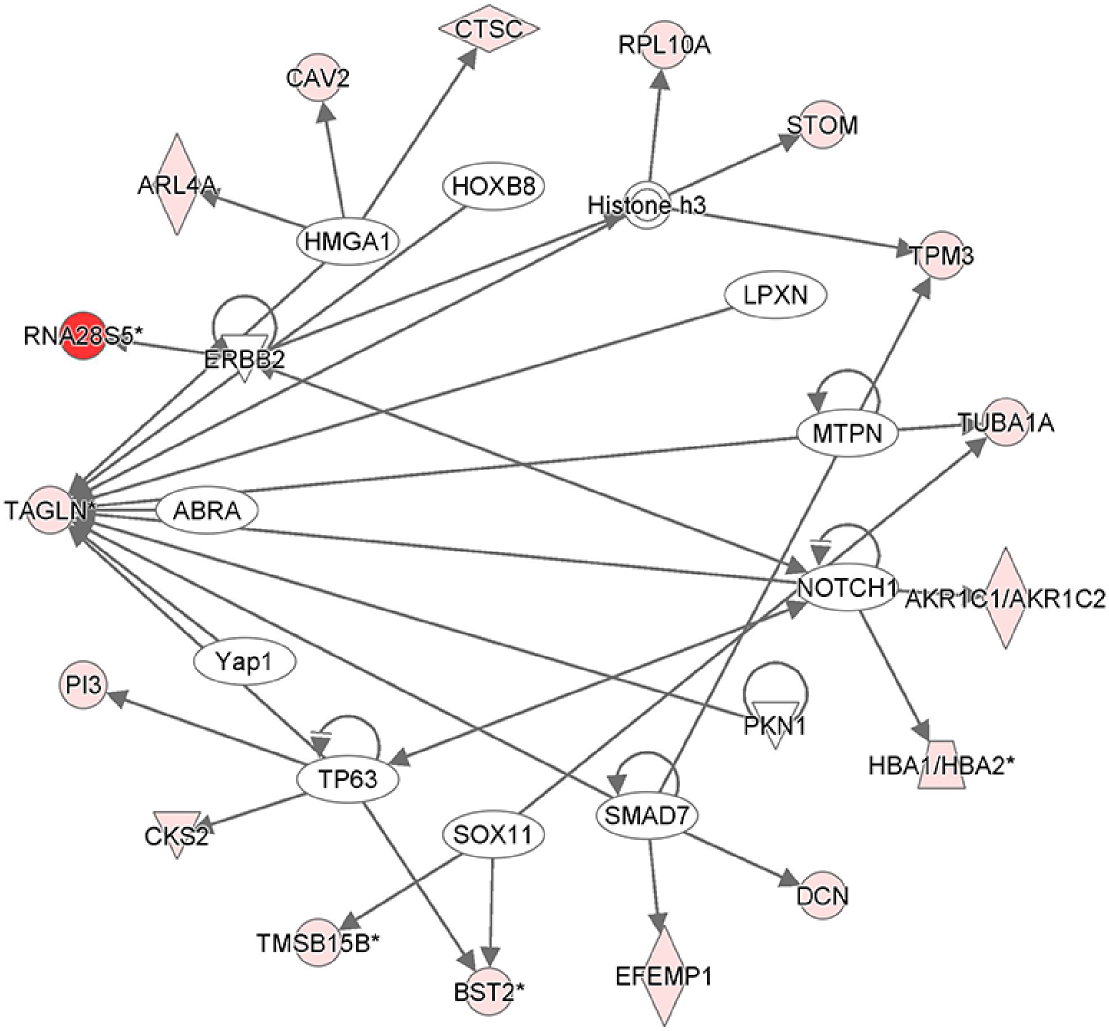

Supplementary Figure 3. Upstream analysis, using IPA, reveals ERBB2 as one of the hubs.
